# Supplementary material for: Transcription Factors in Aureobasidium spp.: Classification, Regulation and a Newly Built Database
Source: J Fungi (Basel). 2022 Oct 17;8(10):1096. doi: 10.3390/jof8101096 (PMC9605165; doi:10.3390/jof8101096)
Supplement: Supplementary file 1 [file jof-08-01096-s001.zip › Table S1.pdf]

Supplementary Table:

Table S1. Accession numbers of the *Aureobsidium* genome sequences included in this study.

Organism	Strain	Assembly accession	Isolation habitat	Sampling site location	Reference
Aureobasidium pullulans	EXF-11900	GCA_004917105.1	Indoors: kitchen refrigerator rubber seal	Croatia: Malinska, Krk	
Aureobasidium pullulans	EXF-11318	GCA_004917135.1	Plant: apple surface	Slovenia: Horjul	
Aureobasidium pullulans	EXF-11319	GCA_004917115.1	Plant: apple surface	Slovenia: Horjul	
Aureobasidium pullulans	EXF-11991	GCA_004917145.1	Indoors: kitchen refrigerator condensation water outlet	Slovenia: Zagorje ob Savi	
Aureobasidium pullulans	EXF-11825	GCA_004917155.1	Indoors: kitchen freezer rubber seal	Slovenia: Bistrica ob Sotli	
Aureobasidium pullulans	EXF-11014	GCA_004917165.1	Plant: commercial biocontrol strain	N.D.	
Aureobasidium pullulans	EXF-11013	GCA_004917185.1	Plant: commercial biocontrol strain	N.D.	
Aureobasidium pullulans	EXF-10751	GCA_004917205.1	Other: cloud sample	France	20
Aureobasidium pullulans	EXF-11323	GCA_004917225.1	Plant: sweet chestnut leaf surface	Slovenia: Horjul	
Aureobasidium pullulans	EXF-10796	GCA_004917255.1	Plant: persimmon surface	Slovenia	
Aureobasidium pullulans	EXF-10659	GCA_004917275.1	Indoors: indoor air sample	Slovenia: Celje	
Aureobasidium pullulans	EXF-10632	GCA_004917305.1	Other: car diesel reservoir inlet inner surface	Slovenia: Jezero	
Aureobasidium pullulans	EXF-10629	GCA_004917595.1	Other: car petrol reservoir inlet inner surface	Slovenia: Jezero	
Aureobasidium pullulans	EXF-10507	GCA_004917335.1	Other: marble block surface	Italy: Messina	
Aureobasidium pullulans	EXF-10085	GCA_004917355.1	Indoors: kitchen cutting board surface	Slovenia: Planina pri Sevnici	
Aureobasidium pullulans	EXF-10081	GCA_004917375.1	Indoors: kitchen sink drain	Slovenia: Ljubljana	
Aureobasidium pullulans	EXF-10080	GCA_004917605.1	Indoors: kitchen sink drain	Slovenia: Ljubljana	

Organism	Strain	Assembly accession	Isolation habitat	Sampling site location	Reference
Aureobasidium pullulans	EXF-9785	GCA_004917385.1	Indoors: Interior of water supply connector	Slovenia: Kapla	
Aureobasidium pullulans	EXF-9399	GCA_004917415.1	Plant: grape surface	Greece: Attica	
Aureobasidium pullulans	EXF-8828	GCA_004917425.1	Glacial: glacial meltwater	Argentina: San Carlos de Bariloche	
Aureobasidium pullulans	EXF-8128	GCA_004917435.1	Plant: maple leaf surface	Slovenia: Ljubljana	
Aureobasidium pullulans	EXF-8127	GCA_004917445.1	Indoors: surface of metal bucket used for carrying water	Slovenia: Cuber	
Aureobasidium pullulans	EXF-6604	GCA_004917485.1	Plant: roots of *Juncus trifidus*	Poland: Babia Góra massif	
Aureobasidium pullulans	EXF-6519	GCA_004917495.1	Other: felt on the bottom side of a metal roof tile	Slovenia: Mengeš	
Aureobasidium pullulans	EXF-6514	GCA_004917505.1	Plant: peach bone	Slovenia	
Aureobasidium pullulans	EXF-6298	GCA_004917525.1	Indoors: washing powder tray	Slovenia: Postojna	
Aureobasidium pullulans	EXF-8841	GCA_004917615.1	Plant: Nothofagus pumilio leaf surface	Argentina: San Carlos de Bariloche	
Aureobasidium pullulans	EXF-8126	GCA_004917625.1	Indoors: metal surface, basement of pumpkin seed oil pressing facility	Slovenia: Gibina	20
Aureobasidium pullulans	EXF-6267	GCA_004917665.1	Hypersaline: salpan evaporating sea water	Slovenia: Sečovlje	
Aureobasidium pullulans	EXF-5628	GCA_004917685.1	Indoors: rubber seal	Slovenia: Blejska Dobrava	
Aureobasidium pullulans	EXF-4010	GCA_004917705.1	Glacial: glacial ice with sediment	Norway: Ny-Ålesund	
Aureobasidium pullulans	EXF-4256	GCA_004917555.1	Glacial: glacial ice	Norway: Ny-Ålesund	
Aureobasidium pullulans	EXF-3863	GCA_004917725.1	Hypersaline: salpans crystalization pond water	Slovenia: Sečovlje	
Aureobasidium pullulans	EXF-3844	GCA_004917745.1	Plant: dried olives	Slovenia	
Aureobasidium pullulans	EXF-3984	GCA_004917795.1	Glacial: glacial ice with sediment	Norway: Ny-Ålesund	
Aureobasidium pullulans	EXF-1645	GCA_004917825.1	Glacial: glacial ice	Norway: Ny-Ålesund	

Organism	Strain	Assembly accession	Isolation habitat	Sampling site location	Reference
Aureobasidium pullulans	EXF-1668	GCA_004918575.1	Glacial: glacial ice from sea water	Norway: Ny-Ålesund	20
Aureobasidium pullulans	EXF-3519	GCA_004918105.1	Plant: oak leaf surface	Slovenia: Ljubljana	
Aureobasidium pullulans	EXF-3403	GCA_004918115.1	Indoors: fourth block wall surface	Ukraine: Chernobyl	
Aureobasidium pullulans	EXF-3374	GCA_004918145.1	Plant: grape surface	France: Beaujeu	
Aureobasidium pullulans	EXF-3380	GCA_004918535.1	Plant: oak slime flux	Germany: Ohlsdorf, Hamburg	
Aureobasidium pullulans	EXF-3358	GCA_004918165.1	Other: sea water	Croatia: Mljet	
Aureobasidium pullulans	EXF-676	GCA_004918195.1	Indoors: air conditioner grate for entering air	Slovenia: Ljubljana	
Aureobasidium pullulans	EXF-674	GCA_004918505.1	Indoors: air conditioner grate for entering air	Slovenia: Ljubljana	
Aureobasidium pullulans	EXF-2618	GCA_004918245.1	Plant: grape surface	Slovenia: Ljubljana	
Aureobasidium pullulans	EXF-3645	GCA_004918275.1	Glacial: glacial ice at the edge of glacier	Norway: Ny-Ålesund	
Aureobasidium pullulans	EXF-3670	GCA_004917815.1	Glacial: glacial ice at the edge of glacier	Norway: Ny-Ålesund	
Aureobasidium pullulans	EXF-3750	GCA_004918215.1	Glacial: glacial ice at the edge of glacier	Norway: Ny-Ålesund	
Aureobasidium pullulans	EXF-3780	GCA_004917755.1	Hypersaline: microbial mat, bottom of the sea water evaporation pond	Puerto Rico: Candelaria	
Aureobasidium pullulans	EXF-150	GCA_000721785.1	Hypersaline waters of the Sečovlje solar saltern	Slovenia	33
Aureobasidium namibiae	CBS147.97	GCA_000721765.1	Dolomitic marble in the Namib Desert	Namibia	
Aureobasidium subglaciale	EXF-2481	GCA_000721755.1	Subglacial ice of the Kongsvegen glacier on Spitsbergen	Svalbard, Norway	
Aureobasidium melanogenum	CBS110374	GCA_000721775.1	Public fountain in Bangkok	Thailand	
Aureobasidium melanogenum	P16	GCA_019915885.1	Leaf of *Acanthus ilicifolius* in mangrove	Dongzhaigou, Hainan Province, China	35
