# Supplementary material for: Transcription Factors in Aureobasidium spp.: Classification, Regulation and a Newly Built Database
Source: J Fungi (Basel). 2022 Oct 17;8(10):1096. doi: 10.3390/jof8101096 (PMC9605165; doi:10.3390/jof8101096)
Supplement: Supplementary file 1 [file jof-08-01096-s001.zip › Table S3.pdf]

Supplementary Table:

Table S3. Major zinc finger transcriptional factors in *Aureobasidium* spp.

Fold Group	PFAM
Class I: Zinc knuckle (zf-CCHC)	PF00098
Class II: Cys2His2 (‘classic zinc finger’)	PF00096
Class III: Zinc finger, C4 type (two domains)	PF00105
Class IV: Fungal Zn(2)-Cys(6) binuclear cluster domain	PF00172
Class V: Putative zinc finger motif, C2HC5-type	PF06221
Class VI: Zinc finger, C3HC4 type (RING finger)	PF00097
Class VII: TF_Zn_Ribbon (TFIIB zinc-binding)	PF08271
