# Supplementary material for: Transcription Factors in Aureobasidium spp.: Classification, Regulation and a Newly Built Database
Source: J Fungi (Basel). 2022 Oct 17;8(10):1096. doi: 10.3390/jof8101096 (PMC9605165; doi:10.3390/jof8101096)
Supplement: Supplementary file 1 [file jof-08-01096-s001.zip › Table S5.pdf]

Supplementary Table:

Table S5. The TFs numbers of *Aureobasidium* spp. in the NCBI (GenBank) and the ATFDB databases.

Type	NCBI (GenBank)	AUREOBASIDIUM TFs
General TFs	2511	322
Global TFs	483	1386
Specific TFs	126	697
