# Supplementary figures and images for: Transcription Factors in Aureobasidium spp.: Classification, Regulation and a Newly Built Database
Source: J Fungi (Basel). 2022 Oct 17;8(10):1096. doi: 10.3390/jof8101096 (PMC9605165; doi:10.3390/jof8101096)

## Supplementary Figure:

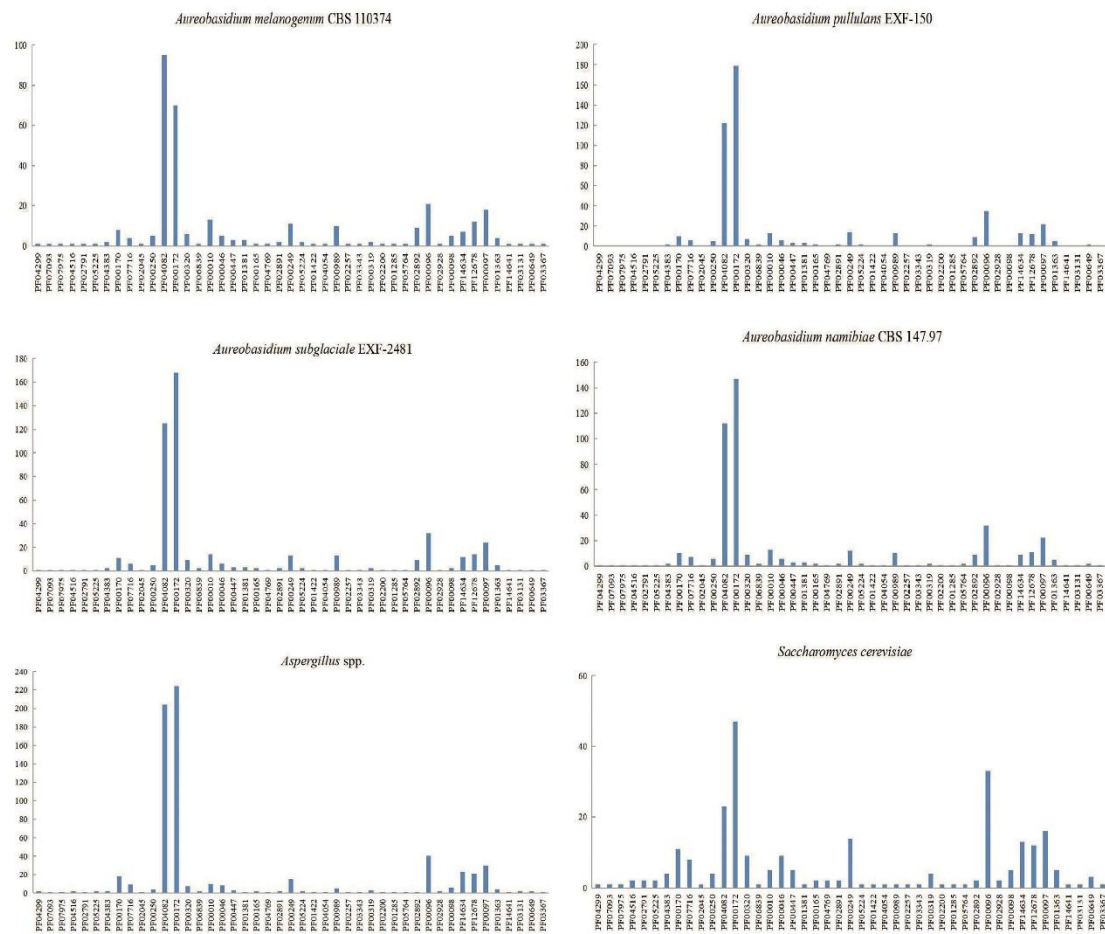

**Figure S1.** The numbers of the PFAM families in different fungi.

Supplement: Supplementary file 1 [file jof-08-01096-s001.zip › Figure S1 and S2.pdf]
